# The Interplay Between Language Form and Concept During Language Switching: A Behavioral Investigation

**DOI:** 10.3389/fpsyg.2020.00791

**Published:** 2020-04-30

**Authors:** Yong Zhang, Ningning Cao, Chang Yue, Lina Dai, Yan Jing Wu

**Affiliations:** ^1^School of Foreign Languages, Southwest University of Political Science and Law, Chongqing, China; ^2^College of International Studies, Southwest University, Chongqing, China; ^3^School of English Studies, Dalian University of Foreign Languages, Dalian, China; ^4^Ningbo Yongjiang Vocational High School, Ningbo, China; ^5^Faculty of Foreign Languages, Ningbo University, Ningbo, China

**Keywords:** language form switch, concept switch, switch costs, switch asymmetry, multiple processing stages

## Abstract

Language switching involves multiple processing stages. Previous studies have not dissociated the cognitive process underlying language form switches and concept switches. Here, we examined the two factors using a novel language-switching paradigm. Chinese-English bilinguals named individually presented pictures in either Chinese or English according to a language cue. Pictures in two consecutive trials represented either identical, semantically related, or unrelated concepts. Results showed both language (form) switch costs and concept switch costs. The interaction between these two factors suggested that the effects were additive, with the longest naming response times observed when two pictures were semantically unrelated and involved a switch between languages. These findings suggest that the functional loci of the language control mechanism occur at multiple processing stages. Implications of the findings are discussed within current models of language processing in bilinguals.

## Introduction

In daily life, bilingual speakers need to switch between the native language and second language according to the interlocutors. The flexibility and efficiency to select the appropriate language depends on the language control mechanism (for reviews see Abutalebi and Green, 2008; Kroll et al., 2008; Declerck and Philipp, 2015a). Experimental Psychology has used the language-switching paradigm to investigate the underlying mechanism of language control during speech production. However, language control may occur at different processing stages (Declerck and Philipp, 2015a). For example, when bilingual speakers switch from the English word “chair” to the Chinese word “
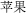
” (“apple”), it requires switching between language forms (i.e., from English to Chinese) and updating concepts (i.e., from “CHAIR” to “APPLE”) (Zhang et al., 2019). To date, the underlying mechanism of language control at multiple processing stages within the language processing system remains an open issue. The present study aimed to address this issue by examining the interplay between language form and concept^1^ during language switching.

### Switch Costs, Mixing Costs, and the Inhibitory Control Model

The language switching paradigm has been extensively used to study language control in bilingual speech production (e.g., Meuter and Allport, 1999; Costa and Santesteban, 2004). In this paradigm, a visual stimulus (e.g., a digit or a picture) is presented following a language cue which indicates the language in which the stimulus needs to be named. Within a block, participants may either switch from one language to another (i.e., switch trials) or repeat the same language as the prior trial (i.e., non-switch trials). As compared to non-switch trials, switch trials usually result in longer naming response times and higher error rates. The differential performance between these two trial types is taken as the “switch costs” (Meuter and Allport, 1999; Costa and Santesteban, 2004; Christoffels et al., 2007; Philipp et al., 2007; Verhoef et al., 2009). Interestingly, switch costs are often reported to be asymmetric in unbalanced bilinguals with a smaller forward (L1-L2, i.e., from the native language to the second language) switch costs as compared to the backward (L2-L1) switch costs (for a review see Bobb and Wodniecka, 2013).

According to the inhibitory control (IC) model (Green, 1998), switch costs entail a transient, trial-by-trial inhibitory mechanism, which resolves between-language interference by suppressing lexical representations of the non-target language, allowing efficient word production in the intended language. Green (1998) suggested that this inhibitory process incurs switch costs. The IC model also predicts switch cost asymmetry with the assumption that the magnitude of inhibition varies as a function of language proficiency. For unbalanced bilinguals, more inhibition is required to suppress activations of the dominant language (i.e., the first or native language or L1) as compared to the weaker language (i.e., the second language or L2) during speech production. Therefore, switching from L1 to L2 is easier as compared to switching from L2 to L1 (for a review see Bobb and Wodniecka, 2013).

Besides switch costs, mixing costs are the other behavioral indicator of the language control process in bilingual speech production. Mixing costs refer to the reduced performance (i.e., longer naming response times and higher naming errors) in non-switch trials of a mixed-language block as compared to that of a single-language block (e.g., Christoffels et al., 2007; Gollan and Ferreira, 2009; Wang et al., 2009; Prior and Gollan, 2013; Ma et al., 2016). Switch costs and mixing costs are assumed to measure different processes of language control, with the former reflecting transient, trial-to-trial inhibitory processes and the latter reflecting sustained and global effect of between-language interference on bilingual speech production (for a review see Kiesel et al., 2010).

### Functional Locus of the Language Control Mechanism and Relevant Studies

The IC model (Green, 1998) explains switch costs with the notion of reactive inhibition of the non-target language. According to the IC model, inhibition occurs at two functional processing stages: the schema and lemma stages. The schema is proposed to control for language interference, a process that is similar to those involved in general cognitive control tasks, such as the nonverbal task-switching paradigm (Cepeda et al., 2000; Barac and Bialystok, 2012), in which participants have to stay focused on the target task (e.g., shape) and ignore the non-target task (e.g., color). This process is outside of language processing. The locus of language control at the lemma stage, which involves the trial-by-trial inhibitory process, is assumed to be within language processing. In contrast to the account of inhibitory mechanism, non-inhibition-based models propose that no inhibition occurs during language switching (e.g., Costa et al., 1999; La Heij, 2005; Philipp et al., 2007). For example, in the activation process model (Philipp et al., 2007), proposed that language control in bilinguals is achieved through facilitation of the target language representations as compared to that of the non-target language.

The functional loci of the language control mechanism can occur at different language processing stages (Kroll et al., 2006; Bobb and Wodniecka, 2013; Gollan et al., 2014). However, the hypothesis that language control might occur at multiple processing stages within language processing has not been systematically studied. For example, the role of concept in language control has prompted some concerns. While several models have proposed a critical role for the concept (Poulisse and Bongaerts, 1994; La Heij, 2005; Schwieter and Sunderman, 2008; Declerck and Philipp, 2015a), little empirical research has been conducted to examine language control at this stage (for comprehension studies see Chee et al., 2003; Crinion et al., 2006; Klein et al., 2006). To our best knowledge, only three studies on bilingual speech production have investigated language control at the stages of both language form and concept (Declerck et al., 2013, 2015; Declerck and Philipp, 2015b; Zhang et al., 2019).

Declerck et al. (2013) used a sequence-based language switching (SBLS) paradigm to examine whether predictable responses to switches in language form and concept would affect the switch costs. Bilingual participants produced words in pre-defined orders (i.e., weekdays, numbers) or a novel sequence and alternated the language after every second trial (e.g., L1-L1-L2-L2-L1-L1). The advantage of this paradigm is that, as both the language sequence and the concept sequence were pre-defined (or pre-learned), neither language cues nor visual stimuli were needed. The results showed switch costs in both language form and concept switch conditions, suggesting the involvement of these two factors in language control (for the same SBLS paradigm see Declerck et al., 2015). However, as the language form and concept sequence were pre-defined, it is debatable whether the effects observed in the SBLS paradigm could be taken as evidence for language control in general. For instance, words such as weekdays (Monday, Tuesday, Wednesday etc.) and numbers (one, two, three etc.) only represent a small part of the lexical memory and speech production in everyday life.

A recent study on language switching investigated the independent effects of language form and concept in bilingual speech production (Zhang et al., 2019). As language switching involves simultaneous switches in language form and semantic concept, Zhang et al. (2019) manipulated these two factors by using a dual-stimuli picture-naming task. In one trial, two pictures were named using either the same language (i.e., language non-switch condition) or different languages (i.e., language switch condition). Also, the same picture (i.e., concept non-switch condition) or different pictures (i.e., concept switch condition) are presented in one trial. A significant interaction between language form and concept was observed in the naming RTs, suggesting that both factors have an impact on language switching. However, in a block design experiment, a language switch is more predictable as compared to a concept switch (participants cannot predict the concept of the picture in a concept switch trial). The results may be confounded by predictability.

### The Present Study

The present study aims to examine how language form and concept modulate language switching during bilingual speech production. The dissociation between language form and concept will allow us to better understand how language control is achieved in bilinguals. Previous studies (e.g., Meuter and Allport, 1999; Costa and Santesteban, 2004; Christoffels et al., 2007; Philipp et al., 2007; Verhoef et al., 2009) have taken language switch as a single cognitive process (i.e., without dissociating language form and concept). However, in real-life circumstances, when bilinguals switch between languages, the content (i.e., concept) of their speech may change or not change; the concepts they express may be semantically related or unrelated. Therefore, to fully examine how speech production is control in bilinguals, we manipulated, independently, language form switch and language concept switch, which includes three levels: repeated concept, semantically repeated concepts, and unrelated concepts.

We modified the standard cued-naming paradigm so that manipulations of both language form and concept are unpredictable. In a 2 × 3 × 2 within-subject design, language switching (switch vs. non-switch), concept switching (repeated vs. related vs. unrelated), and naming language (L1 vs. L2) were manipulated. Pictures in two consecutive trials were either named in the same language (i.e., language non-switch) or different languages (i.e., language switch). The concepts of pictures in two consecutive trials were either (1) identical (semantically repeated), (2) belong to the same semantic category (semantically related. e.g., chair-table), or (3) belong to two different semantic categories (semantically unrelated. e.g., chair-apple). Trials were randomly assigned to four mixed language blocks. Additionally, two single language blocks were included to examine mixing costs. Participants named each picture in either Chinese (L1) or English (L2) according to the cue.

This design allowed us to dissociate language (form) switching from concept switching. Teasing apart these two types of switch costs will help better understand the locus/loci of the language control mechanism. The hypothesis is that if both language form and concept contributed to switch costs, the main effects of these two variables would be expected. If only language form contributed to switch costs, then we would expect language switch costs but no concept switch costs. Moreover, if the magnitude of involvement were different between the two forms of control, an interaction between the two variables would be expected. Based on previous studies (e.g., Christoffels et al., 2007; Prior and Gollan, 2013; Ma et al., 2016), we also expect to observe mixing costs when non-switch trials in mixed language contexts are compared to trials in single language contexts, and switch cost asymmetry between forward (L2 to L1) and backward (L1 to L2) switches (for a review see Bobb and Wodniecka, 2013).

## Materials and Methods

### Participants

Thirty-nine Chinese-English bilinguals (mean age 22 ± 2.19, range 18–26 years old, 34 female) gave written consent to participate in this study. Participants started learning English as their second language (L2) between the ages of 9 and 12 in school. Participants were right-handed (Oldfield, 1971), had normal or corrected-to-normal vision, and reported no language, hearing or neurological impairments. The study was approved by the Ethics Review Board of Southwest University of Political Science and Law, China. Data of one additional participant were not analyzed due to technical failure of the voice recording device.

Prior to the experiment, subjective and objective measures were collected to assess participants’ language proficiency and language use. Subjective measures were obtained through participants’ self-report scores of language proficiency and age of acquisition (AoA) by using the Chinese version of the Language Experience and Proficiency Questionnaire (LEAP-Q) (Marian et al., 2007). The objective measure is a verbal fluency task (animal naming in 60 seconds). Results of both self-ratings and the verbal fluency task were significantly higher in Chinese as compared to English (all *p* < 0.001), suggesting that the participants were unbalanced Chinese-English bilinguals (see Table 1).

**TABLE 1:** Language background of participants: age of acquisition (AoA), scores of self-rated proficiency (0 as the minimum and 10 as the maximum) and the verbal fluency task.

	L1 (Chinese)	L2 (English)
*AOA*		11(2.89)
**Self-rating proficiency**		
Listening (0–10)	9.05(0.99)	5.20(1.60)
Speaking (0–10)	9.05(0.85)	5.41(1.64)
Reading (0–10)	9.07(0.88)	6.91(1.54)
Writing (0–10)	8.51(1.08)	5.94(1.63)
Overall proficiency (0–40)	35.68(3.80)	23.46(6.41)
*Verbal fluency (60 s)*	20.97(3.77)	14.51(2.43)

*Values within parentheses are standard deviations.*

### Stimuli

Forty-eight black-and-white line drawings were selected from Snodgrass and Vanderwart (1980) as the naming stimuli (see Supplementary Material). These experimental pictures were initially screened from 60 pairs of semantically related words out of 120 object pictures. An independent cohort of 40 undergraduates assessed the semantic relatedness of these word pairs with a 5-point scale (0 as unrelated and 5 as mostly related). Based on the screening of semantic relatedness, we selected 24 pairs of semantically related stimuli (semantic relatedness = 3.57, *SD* = 1.14) and 24 pairs of unrelated stimuli (semantic relatedness = 0.54, *SD* = 0.16), independently. The semantic relatedness was significantly different between related and unrelated conditions (*p* < 0.001). The objects depicted in the pictures include common things such as animals, body parts, and fruits.

### Procedure

After providing informed consent, participants familiarized themselves with the pictures and the names of each picture through a paper booklet to reduce the number of errors in the picture-naming experiment. Prior to the experiment, there were practice trials to familiarize the participants with the tasks (8 trials of single-language naming, 4 in Chinese and 4 in English, and 8 trials of mixed-language naming) with pictures that were not included in the actual experiment. The experiment with a total of 960 experimental trials consisted of four single-language blocks, which were presented first, and four mixed-language blocks, which were presented after the single-language blocks. The language(s) of instruction was consistent with the language(s) of the block: instructions were provided in Chinese for the Chinese blocks, English for the English blocks, and in both Chinese and English for the mixed-language blocks. There was a 1 min break between blocks. The first two single-language blocks were short blocks, with each containing 48 trials (each experimental picture presented once). The language of the first two single-language blocks was counterbalanced across participants. The second two single-language blocks were long blocks, with each containing 144 trials. Each experimental picture was presented three times, once in the repeated condition, once in the semantically related condition, and once in the unrelated condition in a pseudo-randomized order. The order of languages in the second two single-language blocks was also counterbalanced across participants.

The four mixed-language blocks contained the same structure as those two long single-language blocks with regard to the concept manipulations (i.e., repeated, semantically related, and unrelated), but the naming language of each trial in the mixed-language blocks varied as a function of the color of the frame (i.e., the language cue) in which the picture was presented. There were 72 language switch trials and 72 language non-switch trials in each block. There were no more than three consecutive language switch or language non-switch trials.

**FIGURE 1:**
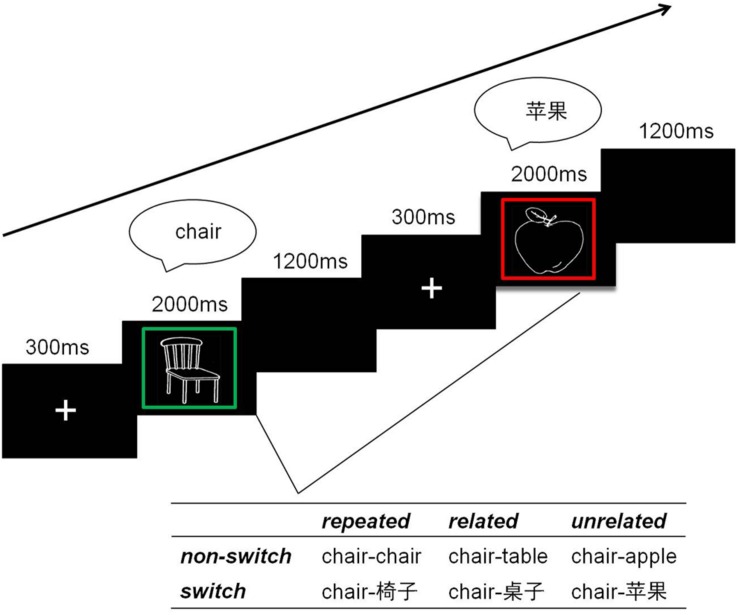
Schematic outline of the experimental trials. In two consecutive trials, the naming language(s) was either the same (non-switch trials: e.g., English-to-English) or different (switch trials: e.g., English-to-Chinese), the concepts were either identical (repeated trials: e.g., CHAIR-to-CHAIR), semantically related (related trials: e.g., CHAIR-to-TABLE) or unrelated (unrelated trials: e.g., CHAIR-to-APPLE).

Each block began with three trials that served as dummy trials for the participants to get started. All trials followed the same pipeline (see Figure 1). In the beginning of a trial, a fixation cross was presented for 300 ms. Following the fixation cross, a picture was presented inside of a colored rectangle frame on a black background. The picture was presented with 300 × 300 pixels and the rectangle frame with 350 × 350 pixels. The participant was instructed to name the picture in either Chinese or English according to the color of the frame. The frame and picture remained on the screen for 2000 ms or disappeared from the screen once the voice key was triggered. When the stimulus disappeared, a blank screen was presented for 1200 ms as the inter trial interval. The colors of the frame included red, blue, yellow, and green. For every participant, each language (Chinese or English) corresponds to two colors so that the color of the cue changed between every two consecutive trials independent of language switches (de Bruin et al., 2018; Jevtović et al., 2019). This manipulation reduced confounds between the cue and language switching (Heikoop et al., 2016). The color-to-language correspondence was counterbalanced across participants. To minimize differences between single-language and mixed-language blocks, all four colors were used in the frames presented in the single-language blocks even though no language switching was needed.

The experiment was conducted on a DELL PC in a dimly lit cabin. The viewing distance was approximately 60 cm from the screen. The experiment was programmed and run using E-prime 2.0. Stimuli and instructions were presented with 90 Hz refresh rate and screen resolution 1024 × 768. Responses were recorded with the Serial Response Box (Psychology Software Tools, Inc.) and a microphone. Errors were coded on the spot by the experimenter. Participants were debriefed at the end of the experiment.

## Results

**FIGURE 2:**
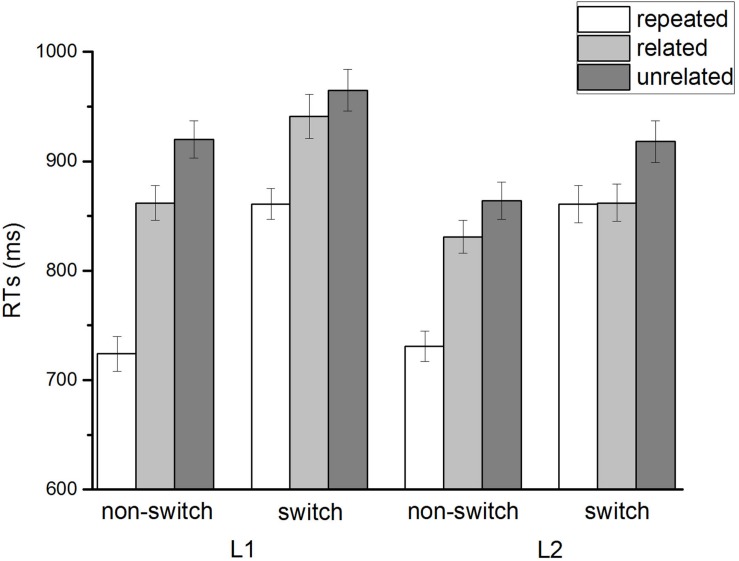
Naming response times (in milliseconds) as a function of language form switching (non-switch and switch), concept switching (repeated, related and unrelated) and naming language (L1 and L2).

For every participant, naming RTs were calculated for correct trials only. A trial was coded as an error if there was no response before the response deadline (i.e., 2000 ms), a wrong answer was given, a wrong naming language was used, or a false start (3.4% of all trials). The first three dummy trials in each block were excluded from further analysis. Naming RTs that were more than 3 standard derivations above or below the individual’s mean value were rejected as outliners. Table 2 shows the mean reaction times (RTs) and mean error rates in each condition (also see Figure 2). As error rates were extremely low across all conditions, error rates were not further analyzed.

**TABLE 2:** Mean reaction times in ms (SD) and error rates (as percentages) presented separately in each condition of the experiment.

	Condition	Mean RT	Mean error rate
	L1 repeated	524(83)	0.004(0.06)
	L1 related	602(93)	0.013(0.11)
Single-language block	L1 unrelated	682(85)	0.015(0.12)
	L2 repeated	553(66)	0.005(0.07)
	L2 related	674(89)	0.012(0.11)
	L2 unrelated	722(79)	0.024(0.15)
	L1 non-switch repeated	724(100)	0.014(0.12)
	L1 non-switch related	862(100)	0.046(0.20)
	L1 non-switch unrelated	920(105)	0.068(0.25)
	L1 switch repeated	861(90)	0.044(0.21)
	L1 switch related	942(125)	0.087(0.28)
	L1 switch unrelated	965(118)	0.077(0.27)
Mixed-language block	L2 non-switch repeated	731(90)	0.012(0.11)
	L2 non-switch related	831(94)	0.023(0.15)
	L2 non-switch unrelated	864(105)	0.022(0.15)
	L2 switch repeated	861(105)	0.054(0.23)
	L2 switch related	862(108)	0.049(0.22)
	L2 switch unrelated	918(117)	0.050(0.22)

The mean RTs in the first two single-language blocks were calculated as a reference and were not included for further ANOVA analysis. Naming in Chinese (729 ms) was significantly faster as compared to naming in English (810 ms) [*t*(38) = 7.04, *p* < 0.001, *r* = 0.71].

Switch costs are defined as the difference in RTs between switch trials and non-switch trials in the mixed-language naming blocks. As language form and concept in the present study are two variables of interest, we dissociated language form switch costs (i.e., switch – non-switch) and concept switch costs (i.e., related, unrelated, and repeated). A three-way repeated measures ANOVA on RTs in the mixed-language blocks was conducted with the naming language (L1, L2), language form switching (non-switch, switch), and concept switching (repeated, semantically related, and unrelated) as within-subject variables. The main effect of the naming language was significant [*F*(1, 38) = 35.42, *p* < 0.001, η^2^ = 0.482], indicating that naming in Chinese (879 ms) was slower than naming in English (844 ms). A reversed language dominance in mixed language blocks was observed with faster naming in L2 than in L1. There was a significant main effect of language form switching [*F*(1, 38) = 146.25, *p* < 0.001, η^2^ = 0.794], indicating that naming was slower in switch trials (901 ms) than non-switch trials (822 ms), with averaged switch costs being 79 ms across conditions. *Post hoc* analysis (paired-wise comparisons, Bonferroni corrected) showed that the effect of language form effect was significant at all three levels of concept switch [i.e., repeated: *t*(38) = 12.86, *p* < 0.001, *r* = 0.76; related: *t*(38) = 6.95, *p* < 0.001, *r* = 0.90; unrelated: *t*(38) = 9.34, *p* < 0.001, *r* = 0.96]. The main effect of concept switching was also significant [*F*(2, 76) = 170.35, *p* < 0.001, η^2^ = 0.902]. *Post hoc* analysis (paired-wise comparisons, Bonferroni corrected) revealed that RTs in the repeated condition (794 ms) were significantly faster than either the semantically related trials (874 ms) [*t*(38) = 13.67, *p* < 0.001, *r* = 0.94] or the semantically unrelated trials (917 ms) [*t*(38) = 18.40, *p* < 0.001, *r* = 0.93], with switch costs being 80 ms and 123 ms, respectively. Further analysis showed that the concept switch effect was comparable in both levels of language form switch (i.e., non-switch: repeated vs. related, *t*(38) = 14.43, *p* < 0.001, *r* = 0.85, repeated vs. unrelated, *t*(38) = 18.60, *p* < 0.001, *r* = 0.84, related vs. unrelated, *t*(38) = 9.88, *p* < 0.001, *r* = 0.96; switch: repeated vs. related, *t*(38) = 6.35, *p* < 0.001, *r* = 0.94, repeated vs. unrelated, *t*(38) = 11.13, *p* < 0.001, *r* = 0.92, related vs. unrelated, *t*(38) = 6.79, *p* < 0.001, *r* = 0.95].

**FIGURE 3:**
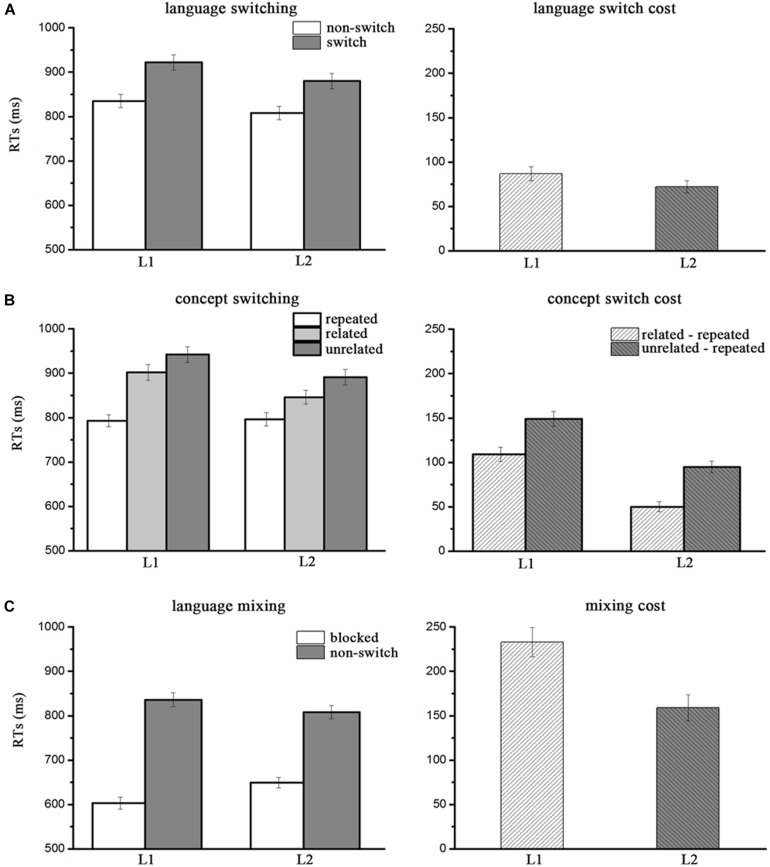
**(A)** Naming response times (in milliseconds; left) and switch costs (right) collapsed by language switch (switch and non-switch) for L1 and L2. **(B)** Naming response times (left) and switch costs (right) as collapsed by concept switching (repeat, related, and unrelated) for L1 and L2. **(C)** Naming response times (left) and mixing costs (right) as collapsed by language context (blocked and non-switch) for L1 and L2. Error bars represent standard errors.

The interaction between language form switching and concept switching was significant [*F*(2, 76) = 44.60, *p* < 0.001, η^2^ = 0.707]. *Post hoc* analysis (paired-wise comparisons, Bonferroni corrected) showed that this interaction was caused by a non-significant comparison between condition in which the two identical pictures were named in different languages (i.e., language form switch) and the condition in which two unrelated pictures were named in the same language [i.e., language form non-switch; *t*(38) = 2.13, *p* = 0.039, *r* = 0.89]. The three-way interaction between naming language, concept switching and language form switching was also significant [*F*(2, 76) = 11.47, *p* < 0.001, η^2^ = 0.383]. Further paired sample *t*-tests showed significant switch costs for both naming in Chinese (87 ms) [*t*(38) = 10.76, *p* < 0.001, *r* = 0.88] and naming in English (72 ms) [*t*(38) = 10.70, *p* < 0.001, *r* = 0.92]. Comparisons between language form switch costs in Chinese and English [*t*(38) = 2.15, *p* = 0.038, *r* = 0.57] revealed that the switch costs were asymmetric, with larger switch costs from English to Chinese as compared to from Chinese to English (see Table 3 and Figure 3A). Similarly, paired sample *t*-tests showed significant switch costs in Chinese for semantically related trials (109 ms) [*t*(38) = 13.60, *p* < 0.001, *r* = 0.89] and for semantically unrelated trials (149 ms) [*t*(38) = 17.75, *p* < 0.001, *r* = 0.88] when compared to the non-switch trials in Chinese. The same analysis obtained significant switch costs in English for semantically related trials (50 ms) [*t*(38) = 8.68, *p* < 0.001, *r* = 0.93] and for semantically unrelated trials (95 ms) [*t*(38) = 14.25, *p* < 0.001, *r* = 0.93] when compared to the non-switch trials. Further analysis showed that concept switch costs were significantly larger in Chinese as compared to in English [*t*(38) = 8.28, *p* < 0.001, *r* = 0.50] (see Table 4 and Figure 3B).

**TABLE 3:** Mean RTs in ms (SD) in language form non-switch and switch trials and switch costs presented separately for Chinese and English.

	Chinese	English
Language form non-switch trials	835 (96)	808 (93)
Language form switch trials	922 (107)	880 (107)
Language form switch costs	87 (50)	72 (42)

**TABLE 4:** Mean RTs in ms (SD) in concept non-switch and switch trials and switch costs presented separately for Chinese and English.

	Chinese	English
Concept repeated trials	793(85)	796(94)
Semantically related trials	902(109)	846(97)
Semantically unrelated trials	942(110)	891(109)
Concept switch costs (related)	109(50)	50(36)
Concept switch costs (unrelated)	149(53)	95(41)
Concept switch costs (collapsed)	129(48)	72(36)

To examine the mixing costs which refer to the difference in RTs between the non-switch trials in the mixed-language blocks and trials in the single-language blocks, we conducted two-way repeated measures ANOVA on RTs with naming language (L1, L2) and language context (blocked vs. mixed) as within-subject factors. The main effect of language context was significant [*F*(1, 38) = 187.63, *p* < 0.001, η^2^ = 0.832]. The results showed that RTs in the single-language blocks (626 ms) were reduced as compared to the mixed-language blocks for non-switch trials (822 ms), indicating mixing costs of 196 ms. The main effect of naming language was not significant [*F*(1, 38) = 3.48, *p* > 0.05, η^2^ = 0.084]. The interaction between naming language and block type was significant [*F*(1, 38) = 37.26, *p* < 0.001, η^2^ = 0.495]. Further paired sample *t*-tests showed significant mixing costs for naming in both Chinese (233 ms) [*t*(38) = 14.30, *p* < 0.001, *r* = 0.360] and English (159 ms) [*t*(38) = 10.78, *p* < 0.001, *r* = 0.404]. The significant difference between the mixing costs in Chinese and English [*t*(38) = 6.10, *p* < 0.001, *r* = 0.699] demonstrated that the mixing costs were asymmetric, with larger costs for naming in Chinese (L1) than naming in English (L2) (see Table 5 and Figure 3C).

**TABLE 5:** Mean RTs in ms (SD) in single- and mixed-language blocks and mixing costs presented separately for Chinese and English.

	Chinese	English
Single-language blocks (blocked trials)	603 (83)	649 (73)
Mixed-language blocks (non-switch trials)	836 (96)	808 (93)
Mixing costs	233 (102)	159 (92)

There was no difference (*p* = 0.274) in L1 naming between participants who named the pictures in L1 first (*M* = 617 ms, *SD* = 103) and those who named the pictures in L1 second (*M* = 588 ms, *SD* = 62). There was also no significant difference (*p* = 0.555) between participants who named the pictures in L2 first (*M* = 639 ms, *SD* = 93) and those who named pictures in L2 second (*M* = 653 ms, *SD* = 52). Moreover, we conducted Pearson correlation analyses and found a significant positive correlation between the cross-language proficiency variance (i.e., L1 overall proficiency vs. L2 overall proficiency) and the magnitude of the switch costs. More L1 dominant bilinguals showed a greater switch cost from L2 to L1 (*r* = 0.407, *p* = 0.010), but not from L1 to L2 (*r* = 0.186, *p* = 0.256), as compared to less dominant bilinguals (e.g., more balanced bilinguals). Variance in verbal fluency between L1 and L2, however, was not significantly correlated with switch cost (*p* = 0.511).

## Discussion

The present study examined the interplay between language form and concept during bilingual speech production. To this end, we developed a novel paradigm which dissociated these two factors. Switch costs, the classic language control index, were analyzed across two language-switching conditions (non-switch and switch) and three concept-switching conditions (repeated, related, and unrelated). Results showed significant switch costs in both language form and concept, with an interaction between these two factors. The findings suggest that the language control system involves not only the control of language form but also the control of concept during bilingual speech production. We will first discuss the findings at the processing stages of language control, taking these two factors independently. Then we will synthesize the discussions in the framework of multiple loci of language control in bilinguals.

### Language Control in Language Form and Semantic Concept

Previous studies on bilingual speech production have often failed to tease apart different processing stages, such as language form and concept, when examining language switching in bilinguals (e.g., Meuter and Allport, 1999; Costa and Santesteban, 2004; Christoffels et al., 2007; Philipp et al., 2007; Verhoef et al., 2009). The present study observed both the concept and language (form) switch costs by dissociating language (form) switch from concept switch.

Language (form) switch costs were examined by comparing language switch condition to non-switch condition. Without a change in concept, language form switch is similar to a translation process (e.g., Hernandez and Kohnert, 2015) and also similar to cross-language switching in Zhang et al. (2019). The IC model (Green, 1998) proposes that language control involves persistent inhibition of the nontarget language. A previously inhibited language will suffer suppression effects when it becomes the target language. The switch costs resemble (negative) carryover effects from one trial to the next trial. In this case, language interference resolution (i.e., inhibition) occurs at the language form stage only, between a word in one language and its lexical equivalent in the other language, but it may not be conceptually mediated (for a review see Snodgrass, 1993) because the concept, which is connected to both the target lemma and its translation-equivalent lemma, remains unchanged.

Language switching usually involves not only language (form) switch but also concept switch (Zhang et al., 2019). In this study, we manipulated concept switch at three levels (i.e., repeated, related and unrelated) independent of language form switch and found concept switch costs in both the semantically related and the unrelated conditions when compared to the repeated condition. The critical role for the concept level in language control has been proposed in several models (Poulisse and Bongaerts, 1994; La Heij, 2005; Schwieter and Sunderman, 2008; Declerck and Philipp, 2015a). For example, La Heij (2005) claimed that the cognitive mechanisms of language control at the conceptual level might involve differently weighed connections to the corresponding lemmas (see also Schwieter and Sunderman, 2008). For instance, when a naming cue is related to the picture at the conceptual level, it raises activation levels of words in the intended language as compared to words in the unintended language, by assigning more “weights” to the connections between the concept and the lemmas in the intended language but not the unintended language.

Moreover, the results showed that related and repeated concepts were facilitated as compared to unrelated concepts. Semantic priming effects have also been observed in monolinguals when performing picture naming tasks (Bloem and La Heij, 2003; La Heij et al., 2003). However, the control mechanism is fundamentally different between monolinguals and bilinguals, as bilingual speakers need to select both the concept and the intended language of speech. While the concept switch effects can be explained by the activation facilitation mechanism proposed by Philipp et al. (2007), at the level of language form, the selection process calls for an inhibitory mechanism, which reduces activation levels of lexical candidates from the unintended language (Green, 1998). The current findings further specify the nature of the language control mechanism, which involves both inhibition and facilitation.

Previous studies have proposed several possible loci of bilingual language control within the language processing system, ranging from the conceptual stage (Poulisse and Bongaerts, 1994; La Heij, 2005), lemma stage (Green, 1998), and phonological stage (Gollan et al., 2014; Declerck and Philipp, 2015c) to orthographical stage (Grainger and Beauvillain, 1987). However, language control may occur at more than one locus (Green, 1998; Declerck and Philipp, 2015a). As shown in the present study and the study by Zhang et al. (2019), there are language form-based control and concept-based control during language switching, suggesting that the language control mechanism in bilinguals has multiple functional loci with one working at the concept level and the other working at the language form level. The notion of switch costs being separate from concept switch and language (form) switch is theoretically significant. It is in line with the idea proposed by most language control models that control during language switching occurs at multiple loci (Green, 1998; Dijkstra and Van Heuven, 2002; Schwieter and Sunderman, 2008; Declerck et al., 2015). In a broader sense, the language control process may occur within language processing and outside of language processing (Green, 1998; Declerck and Philipp, 2015a).

### Language Switching: Mixing Costs, Reversed Language Dominance, and Switch Cost Asymmetry

Mixing costs were observed when naming RTs in the non-switch trials of the mixed-language blocks were compared to the single-language blocks. Consistent with previous studies using cued language naming task, we observed longer RTs in the mixed-language blocks as compared to the single-language blocks (Christoffels et al., 2007; Prior and Gollan, 2011; de Bruin et al., 2018; Peeters and Dijkstra, 2018). Critically, mixing costs were observed in all three conceptual conditions: repeated, semantically related, and unrelated. Mixing costs have been explained as efforts required maintaining and alternating between two languages, reflecting sustained global inhibition during bilingual speech production. de Bruin et al. (2018) showed that mixing costs are related to whether language switching is voluntary or prompted by a cue. In free switch trials, both unbalanced and highly proficient bilinguals showed mixed benefits (i.e., reduced RTs in the mixed-language as compared to single-language condition). In contrast, when the language switch is explicitly cued, there were mixing costs.

The present results also showed reversed language dominance: faster responses when naming in L2 than in L1 in mixed-language blocks. The reversed language dominance has been reported in studies examining unbalanced bilinguals (e.g., Costa and Santesteban, 2004; Christoffels et al., 2007; Gollan and Ferreira, 2009; Verhoef et al., 2009; Peeters et al., 2014; Kleinman and Gollan, 2016; Peeters and Dijkstra, 2018; Wu et al., 2018; Liu et al., 2019; Peeters, 2020). The present study showed a global slowdown of the L1 across semantic conditions in switch trials. The global inhibition of the dominant language has been explained as the result of sustained inhibition required to suppress L1 during L2 naming in mixed-language contexts (Christoffels et al., 2007; Misra et al., 2012). In the present study, the inhibition of the L1 was also observed in repeated trials. Although one would expect faster RTs when naming repeated items in L1 than in L2, naming repeated trials across languages resulted in similar response times, suggesting that even the repeated trials experienced this global L1 suppression.

Consistent with previous studies (e.g., Meuter and Allport, 1999; Philipp et al., 2007; Wang et al., 2007; Verhoef et al., 2009; Macizo et al., 2012; Jin et al., 2014; Peeters et al., 2014), asymmetric switch costs were observed in the present study as language switches were faster from L1 to L2 as compared to from L2 to L1. Asymmetric switch costs have been taken as a behavioral marker for transient local inhibition (for a review see Bobb and Wodniecka, 2013). Interestingly, the same pattern of asymmetry was observed in the mixing costs, with larger mixing costs in the L2 to L1 switches as compared to L1 to L2 switches. Asymmetric mixing costs are taken as the behavioral marker of sustained global inhibition (Christoffels et al., 2007; Bobb and Wodniecka, 2013; Peeters and Dijkstra, 2018). These findings therefore, lend strong support to the IC model of bilingual speech production at both the local and the global control levels. In the same vein, results from the correlational analysis showed that participants with a larger between-language variance in proficiency also showed a greater switch cost asymmetry. As predicted by the IC model, low proficient, unbalanced bilinguals require stronger inhibition to L1 during L2 production, as compared to relatively high proficient bilinguals.

In a previous study (Misra et al., 2012), RTs in L1 naming were reduced when it was performed before, as compared to after, the L2 naming block, indicating a carry-over effect of inhibition on L1 between naming blocks. Interestingly, there was no such effect in the present study. This discrepancy might be due to details in experimental settings. It was not the goal of the present study to examine long-lasting inhibition effects across naming blocks. To the opposite, it was necessary to reduce any carry-over effect between blocks, in order to minimize its influences on within-block performance (switch vs. non-switch). Therefore, the inter-block breaks were relatively long (i.e., 1 min) in the present study. In addition, the number of trials in the single-language naming blocks was relatively small (i.e., 48 trials compared to 72 trials in Misra et al., 2012) in the present study. Finally, in the present study, participants were pre-trained with the picture names, a procedure which may have reduced L1 inhibition required for naming pictures in L2. These variations in experimental parameters might explain the finding that L1 naming did not take longer time when it was performed before or after L2 naming.

The present study differs from the study by Declerck et al. (2013) in at least two aspects. Firstly, different from the predictable language sequence and concept sequence in Declerck et al. (2013), the present study implemented a random language sequence and concept sequence. Secondly, by including a repeated condition, the present study used conceptual repetition as the baseline in comparison to semantic relatedness so that concept switch was an independent variable, whereas Declerck et al. (2013) did not take the concept sequence as an independent variable. The present study also differs from the study by Zhang et al. (2019) in two aspects. First, the language sequence was predictable but the concept sequence was unpredictable in Zhang et al. (2019); whereas neither the language sequence nor the concept sequence was predictable in the present study. Secondly, the present study was concerned with between-trial (trial-to-trial switch) control processes while Zhang et al. (2019) focused on within-trial (switch within a trial) control processes.

## Conclusion

The present study demonstrates that both language form and concept contribute to language switch costs, suggesting that language control occurs at the processing stages of language form and concept during bilingual speech production. This is consistent with the idea that the loci of the language control mechanism occur at multiple stages of processing. These findings help further understand the underlying mechanism of language control. Future research will need to specify factors that determine language control outside of language processing.

## Data Availability Statement

The datasets generated for this study are available on request to the corresponding author.

## Ethics Statement

The studies involving human participants were reviewed and approved by the Ethics Review Board of School of Foreign Languages, Southwest University of Political Science and Law. The patients/participants provided their written informed consent to participate in this study.

## Author Contributions

YZ and YW design the study. NC and CY performed the experiment. YZ, YW, and LD wrote the manuscript.

## Conflict of Interest

The authors declare that the research was conducted in the absence of any commercial or financial relationships that could be construed as a potential conflict of interest.

## Footnotes

1 Here “language form and concept” refers to “lexical form and meaning.” Note that “concept” belongs to the pre-linguistic stage of processing and is often considered non-linguistic specific. But we used the term “language (form) and concept” to keep in line with previous relevant studies (Declerck et al., 2013, 2015; Zhang et al., 2019).
