# Supplementary material for: The Interplay Between Language Form and Concept During Language Switching: A Behavioral Investigation
Source: Front Psychol. 2020 Apr 30;11:791. doi: 10.3389/fpsyg.2020.00791 (PMC7205015; doi:10.3389/fpsyg.2020.00791)
Supplement: Supplementary file 1 [file Table_1.DOC]

**Appendix**

| table(桌子)-chair(椅子) | cat(猫)-mouse(老鼠) | apple(苹果)-orange(桔子) |
| --- | --- | --- |
| bee(蜜蜂)-butterfly(蝴蝶) | arm(胳膊)-leg(腿) | airplane(飞机)-boat(轮船) |
| door(门)-window(窗) | clock(钟)-watch(表) | leaf(叶子)-tree(树) |
| car(汽车)-train(火车) | shirt(衬衫)-skirt(裙子) | chicken(鸡)-dog(狗) |
| ear(耳朵)-mouth(嘴) | sun(太阳)-moon(月亮) | spoon(勺子)-fork(叉子) |
| bread(面包)-cake(蛋糕) | key(钥匙)-lock(锁) | lion(狮子)-tiger(老虎) |
| comb(梳子)-hair(头发) | hand(手)-foot(脚) | television(电视)-fridge(冰箱) |
| pencil(铅笔)-ruler(尺子) | shoe(鞋)-sock(袜子) | cow(牛)-horse(马) |
